# Effects of the Workplace Health Promotion Activities Soccer and Zumba on Muscle Pain, Work Ability and Perceived Physical Exertion among Female Hospital Employees

**DOI:** 10.1371/journal.pone.0115059

**Published:** 2014-12-10

**Authors:** Svein Barene, Peter Krustrup, Andreas Holtermann

**Affiliations:** 1 Faculty of Public Health, Hedmark University College, Elverum, Norway; 2 Department of Sports, University of Nordland, Bodø, Norway; 3 Department of Nutrition, Exercise and Sports, Section of Human Physiology, Copenhagen Centre for Team Sport and Health, University of Copenhagen, Copenhagen, Denmark; 4 Sport and Health Sciences, College of Life and Environmental Sciences, University of Exeter, Exeter, United Kingdom; 5 National Research Centre for the Working Environment, Copenhagen, Denmark; Vanderbilt University, United States of America

## Abstract

**Objectives:**

This 40-week workplace physical training RCT investigated the effect of soccer and Zumba, respectively, on muscle pain intensity and duration, work ability, and rating of perceived exertion (RPE) during work among female hospital employees.

**Methods:**

107 hospital employees were cluster-randomized into two training groups, and a control group. The training was conducted outside working hours as two-three 1-h sessions per week for the first 12 weeks, and continued as one-two 1-h sessions per week for the last 28 weeks. Muscle pain intensity and duration, work ability, and RPE during work were measured at baseline and after 12 and 40 weeks.

**Results:**

After 12 weeks, both the soccer (−1.9, 95% CI, −3.0, −0.8, P = 0.001) and the Zumba group (−1.3, 95% CI, −2.3, −0.3, P = 0.01) reduced the pain intensity (on a scale from 0 to 10) in the neck-shoulder region (eta squared = 0.109), whereas only the soccer group (−1.9, 95% CI, −3.2, −0.7, P = 0.002, eta squared = 0.092) showed a reduction after 40 weeks referencing the control group. After 40 weeks, both the soccer (-16.4 days, 95% CI, −29.6, −3.2, P<0.02) and the Zumba group (-16.6 days, 95% CI, −28.9, −4.2, P<0.01) reduced the pain duration during the past 3 months in the neck-shoulder region (eta squared = 0.077). No significant effects on intensity or duration of pain in the lower back, RPE during work or work ability were found.

**Conclusions:**

The present study indicates that workplace initiated soccer and Zumba training improve neck-shoulder pain intensity as well as duration among female hospital employees.

**Trial Registration:**

International Standard Randomized Controlled Trial Number Register ISRCTN 61986892.

## Introduction

Work within the health care sector is generally characterized by being physically demanding [1]–[3]. Moreover, health care workers are reported to have generally low physical capacities [4].

Such an imbalance between the physical work demands and the capacity of the worker may lead to high rating of perceived exertion during work [5], muscle pain [6]–[9] and reduced work ability, all well known to increase the risk for sick absence [10]–[12] and premature drop-out from the labor market [3].

In theory, improvement in physical capacity should improve perceived exertion during work, muscle pain and work ability among workers with high physical work demands [5], [13]. However, despite previous workplace physical training intervention studies having reported improvements in physical capacity among health workers [4], [14], there is a lack of evidence for corresponding improvements in perceived exertion during work, muscle pain or work ability [15].

Both soccer and Zumba training are considered to be popular physical activities with health beneficial effects among women [16], [17]. Recently, we have performed a randomized controlled trial involving soccer and Zumba training among female hospital employees [14], [18]. We observed improvements in aerobic fitness and fat metabolism of both soccer and Zumba after 12 weeks [14], whereas only the Zumba group showed an improvement in aerobic fitness after 40 weeks. After 40 weeks, both groups maintained the improvements in fat metabolism obtained after 12 weeks [18]. However, it is unknown if soccer and Zumba training offered through the workplace will improve pain in the neck-shoulder and lower back regions, work ability and perceived physical exertion during work.

Therefore, the aim of the present study was to investigate the effects of soccer and Zumba training on muscle pain intensity and duration, work ability and perceived physical exertion during work among female hospital employees. The hypothesis of the study is that both soccer and Zumba training conducted as two-three 1-h sessions per week will decrease musculoskeletal pain in the neck-shoulder- and lower back region, as well as the rating of perceived exertion during work among female hospital employees. Moreover, our hypothesis is that soccer and Zumba training will improve the work ability among the participants.

## Materials and Methods

### Study design

The present study was designed as a cluster-randomized controlled training intervention study conducted among hospital employees aiming to evaluate the effectiveness of two different types of training exercises, i.e. soccer and Zumba. The study was conducted between January 2011 and October 2011, initiated by baseline measurements of anthropometric characteristics, questionnaire containing questions on work-related factors (i.e. occupational seniority, departmental affiliation, etc.), muscle pain intensity and duration, work ability, and perceived physical exertion during work. During the 40 weeks training intervention, follow-up questionnaires were used after 12 and 40 weeks, respectively. The project was ethically approved by the Regional Committees for Medical and Health Research Ethics (REK), Norway (2010/2385–8), and all participants gave their written informed consent (approved by REK) to participate in the study. The project is registered in the International Standard Randomized Controlled Trial Number Register (ISRCTN61986892). The protocol for this trial and supporting CONSORT checklist are available as supporting information; see S1 Checklist and S1 Protocol. Results from the primary outcomes, i.e. physiological health effects from the two different types of physical exercise, are previously published [14], [18]. The present study evaluates the effect on pain, work ability and perceived physical exertion.

### Recruitment of participants

A flow chart of the recruitment of participants is presented in S1 Figure. We aimed for recruiting health care personnel (primarily nurses and healthcare assistants) to this project. An inquiry was therefore directed to the Director of Personnel at a larger Hospital in Norway, consisting of several districts with approximately 3500 employees in total. A district located in the municipality of Bodø, with 961 employees in total, of which 674 worked as nurses or healthcare assistants was selected based on geographical convenience. An information note targeting these 674 employees, of which 548 were females, was published on the Hospital’s intranet. However, due to a request from the manager of the Department of Laboratory Medicine, additional 112 female employees from other professions (mainly bioengineers) were invited. Based on this, a total of 660 female hospital employees were given the opportunity to participate in the study. Three information meetings on different dates and geographical locations were conducted between September 6 and 10, 2010. At the end of the meetings, the attending health personnel were asked to fill out a screening questionnaire, and give consent (consenters) or not (non-consenters) to enroll in the study. For employees who did not attend the information meeting, managers or other colleagues subsequently handed them written information about the project, and screening questionnaires with a stamped addressed envelope to the project manager. Consenters were then invited for physical testing and questionnaire session outside working hours.

A total number of 185 healthcare employees filled out the screening questionnaire. Of these, 147 were nurses or healthcare assistants and 38 employees of other professions, mainly bioengineers and social educators. Due to unforeseen administrative and logistic challenges at the workplace, there was a 10 week delay from filling out screening questionnaire to the baseline test. During this period, 62 dropped-out from the study. After the baseline tests, another 5 employees chose to withdraw.

The criteria for inclusion in the study were hospital employees of either sex, aged 25 to 65 years. Exclusion criteria were pregnancy, diagnosed angina pectoris and life-threatening diseases. A total of 118 persons (107 females and 11 males) fulfilled the inclusion criteria and consented to participate in the study, and were randomly allocated either to a soccer group, a Zumba group or a control group (S1 Figure). The number of participants in the flow chart (S1 Figure) corresponds to the number of participants who were randomized after the baseline test (N = 107), whereas the number of participants in the tables (S1 and S2 Tables) corresponds to the number responders both randomized and answering to the Questionnaire (N = 103).

### Randomization procedure

The randomization procedure was conducted by the project leader, and is previously reported [14]. In short, health care personnel in a single department working in close proximity to each other formed cluster 1 (n = 28), then two almost equal sized clusters, cluster 2 (n = 27) and 3 (n = 29) were matched on sex, BMI, age and work seniority. Information about age and work seniority was obtained from questionnaire. The remaining consenters were then assigned into 3 smaller clusters, cluster A (n = 11), B (n = 11) and C (n = 12), matched on the same variables as above. The randomization was made by lot by blinded staff. The selection was conducted by drawing from 3 boxes; 1) the three different groups (soccer, Zumba or control), 2) the three large clusters (1, 2 or 3), and 3) the three small clusters (A, B or C). The selection was initiated by drawing one group from box 1, followed by drawing one cluster from box 2. This procedure was conducted until each cluster from box 2 was selected into either the soccer, Zumba or control group. The selection of the small clusters was conducted in the same manner as the above, i.e. randomly combined with one of the groups. The group composition was as follows; the soccer group: cluster 3+C, the Zumba group: cluster 2+B and the control group: cluster 1+A [14]. Due to the low number of male participants (n = 11), only females were included in the statistical analyses.

### Intervention content

The training intervention was conducted between January 11 and March 31 in 2011. For both intervention groups, the intervention (training) activities were conducted outside working hours during two-three 1-h sessions per week for 40 weeks. External training facilities and instructors were arranged for both intervention groups.

The soccer group performed soccer sessions consisting of ordinary three-a-side or four-a-side matches in a traditional gymnastics hall (10×20 m) owned by the hospital, and/or five-a-side, six-a-side, seven-a-side matches in a municipal sports hall (20×40 m) located 3 km from the hospital. Each training session was initiated by a 5-min low intensity warm-up period and included a 5-min half-time break. The soccer-sessions were fully supervised by an instructor throughout the 12-week intervention period. Because only a few participants had previous experience with soccer, there was a 30-minutes introduction of basic technical exercises in the first training session.

The Zumba sessions were conducted at a fitness centre located 3 km from the hospital, and consisted of continuous dance-movements to Latin music with varying intensity level throughout the sessions. Each session was initiated with low-intensity movements for the first 5 min, followed by an increasing intensity throughout the workout. At the end of the training session, the intensity was gradually reduced. The Zumba sessions were supervised by 3 certified Zumba instructors, each responsible for regular sessions per week.

### Prior experience and familiarity to the activities

In the soccer group, the participants reported to have little or no previous experience with soccer. In the Zumba group, three of the participants reported to have performed 2–3 sessions of Zumba at a local Fitness Centre prior to the study. The other 36 participants reported no previous experience with Zumba.

### Measurement procedures pre, during and post the intervention

All consenters were invited to several measurements at baseline (between January 3 and 7, 2011), after 12 (follow-up test 1, between April 4 and 15, 2011) and 40 weeks (follow-up test 2, between October 17 and 28, 2011). These measurements included anthropometric data, i.e. body weight (kg) and height (cm) from which the body mass index (BMI) was calculated. The measurements of health and capacity are described in details elsewhere [14], and included e.g. maximal oxygen consumption test (incremental cycle ergometer test), body composition test (DXA-scan) and fasting blood sampling (e.g. plasma osteocalcin, leptin, glucose, cholesterol). The participants were requested not to perform any kind of exhaustive exercise the day before test. The group allocation was concealed for the test leader.

The Nordic Musculoskeletal Questionnaire [19] was used to examine muscle pain intensity and duration in different body regions. Out of the total number of 107 female participants in the study, 103 completed the questionnaire. The questions “please indicate the pain intensity in the [body] region the past 7 days” with response categories on a Likert scale from 0 to 10 (0 = no pain, 10 = maximal pain), and “please indicate the number of days with pain in the [body region] the past 3 months”. The body regions were the neck-shoulder and the lower back, respectively.

Work ability was measured by the single-item from the Work Ability Index (WAI) by Ilmarinen (2004). The questions were; “Please indicate your current work ability compared with lifetime best on a scale from 0 to 10” (0 = completely unable to work, 10 = work ability at its best).

Borg’s Rating of Perceived Exertion (RPE) during work was measured with the question; “Please indicate the level of perceived physical exertion during work on a 15 category scale from 6 to 20” (6 = no exertion at all, 20 = maximal exertion) [20].

### Statistical analyses

Before initiation of the study, a power calculation was carried out for the primary outcome, VO_2_max. Power was set at 0.8 with a significant level of 0.05. Based on previous workplace studies aiming to increase VO_2_max using comparable physical exercise interventions, an average improvement of 5% with a standard deviation of 10 from baseline values was expected. Based on these assumptions, 32 participants were needed for comparison between each respective intervention group and the control group. Calculations of intra-cluster correlation coefficient were conducted on all outcome variables and revealed a modest within subject correlation with p ranging from 0.2 to 0.6. The model assumptions for applying analyses of covariance (ANCOVA) (i.e. normal distribution of outcome variables, correlations between covariates, homogeneity of regression and linearity) were evaluated before performing the statistical analyses. For evaluating the effectiveness of the intervention, ANCOVA analyses with adjustment for baseline values of BMI and cluster affiliation were performed in accordance to the intention-to-treat (ITT) principle, i.e. all randomized participants were included in the analyses, with missing values substituted using standard carry forward or backwards procedures [21]. BMI was considered to be a potential confounder due to a modest difference in baseline BMI between one of the intervention groups and the control group, whereas cluster affiliation was used to limit any intra cluster correlation bias. To avoid false negative results, further explorative analyses were performed using the ANCOVA model with BMI as covariate including data from only the participants who completed both the pre and post-tests (defined as per protocol analyses). All results are given as contrast estimates between the intervention groups and the control group, respectively, together with an associated confidence interval and level of significance. *P*<0.05 is defined as level of statistical significance, and is based on the estimated marginal means with BMI as covariate. The Bonferroni method correcting for multiple statistical tests was used for post hoc-analyses. IBM SPSS Statistics version 22 was used for all statistical analyses.

## Results

### Baseline characteristics of the population

The average age, weight, height, body mass index (BMI) and work seniority were 45.8±9.3 yrs, 70.6±9.7 kg, 166.9±5.9 cm, 25.3±3.1 kg/m^2^, and 78±64 months, respectively (S1 Table). Average degree of pain intensity on a scale from 0 to 10 in the neck-shoulder region and the lower back region was 2.0±2.1 and 1.5±1.9, respectively. Furthermore, the average work ability on a scale from 0 to 10 was 8.4±1.3 (S1 Table). No significant between-group differences were observed at baseline.

### Adherence to the intervention

In the present study, 72 female consenters were randomized to the two training groups, i.e. 37 in the soccer group (SG) and 35 in the Zumba group (ZG). Of these, 6 participants (SG: 4/ZG: 2) chose to withdraw prior to the intervention. Of the remaining 66 who started participating in the training intervention, 10 participants (15%, SG: n = 7/ZG: n = 3) stopped training after 6 weeks. Based on this, 56 participants (84%, SG: n = 26/ZG: n = 30) completed intervention period 1 (1–12 weeks). However, 2 out of the 7 participants who dropped out from training in the soccer group chose to complete the post-test after 12 weeks. Out of the remaining 56 participants, 18 participants (27%, SG: 12/ZG: 6) chose to withdraw prior to intervention period 2. A total of 38 participants (58%, SG: n = 14/ZG: n = 24) started up with training in intervention period 2 (12–40 weeks). Of these, 7 participant (11%, SG: n = 2/ZG: n = 5) dropped out before 25 weeks, whereas 5 participants (8%, SG: n = 1/ZG: n = 4) dropped out between 25 and 35 weeks. From this, a total of 26 participants (39%, SG: n = 11/ZG: n = 15) completed the 40 weeks intervention period.

### Questionnaire response rates

With regard to the questionnaire, 102 out of 107 (95%) completed the questionnaire at baseline. The corresponding response rates after 12 and 40 weeks were 90 (84%) and 69 (64%) completers, respectively.

### Muscle pain

Based on intention-to-treat analyses (ITT), both the soccer group (P = 0.001) and the Zumba group (P = 0.01) significantly decreased pain intensity (on a scale from 0 to 10) in the neck-shoulder region after 12 weeks compared to the control group. The magnitude of the differences in the means was moderate for both intervention groups, i.e. −1.9, 95% CI, −3.0, −0.8 in the soccer group vs. −1.3, 95% CI, −2.3, −0.3 in the Zumba group, respectively (eta squared = 0.109) (S2 Table). After 40 weeks, the soccer group showed a significant decrease (P = 0.002) in the pain intensity in the neck-shoulder region compared to the control group (−1.9, 95% CI, −3.2, −0.7), which corresponds to a moderate effect size (eta squared = 0.092). No such decrease was observed for the Zumba group compared to the control group (−0.9, 95% CI, −2.0, 0.3, P = 0.13) (S2 Table).

With respect to pain intensity in the lower back-region, no significant change was observed for the intervention groups compared to the control group neither after 12 or 40 weeks (S2 Table).

### Duration of muscle pain

After 12 weeks, both the soccer group (-9.2 days, 95% CI, −20.0, 1.6, P<0.10) and the Zumba group (-7.0 days, 95% CI, −17.2, 3.1, P = 0.17) showed a numerical, but insignificant reduction in number of days with pain in the neck-shoulder region the past 3 months compared to the control group (S2 Table). However, after 40 weeks both the soccer group (P<0.02) and the Zumba group (P<0.01) significantly decreased the number of days with pain in the neck-shoulder region compared to the control group. The magnitude of the differences in the means was moderate for both intervention groups, i.e. -16.4 days, 95% CI, −29.6, −3.2 in the soccer group vs. -16.6 days, 95% CI, −28.9, −4.2 in the Zumba group, respectively (eta squared = 0.077).

With regard to number of days with pain in the lower back during the past 3 months, no significant change were observed neither for the soccer group nor the Zumba group compared to the control group (S2 Table).

### Work ability

After 12 weeks, the Zumba group tended to increase (P = 0.14) the work ability (on a scale from 0 to 10) compared to the control group. The magnitude of the differences in the means was moderate, i.e. 0.5, 95% CI, −0.2, 1.1 (eta squared = 0.029), with no such increase in the soccer group compared to the control group (0.1, 95% CI, −0.6, 0.8, P = 0.84). After 40 weeks, no significant change in work ability was observed in either of the training groups compared to the control group.

### Rating of perceived exertion (RPE) during work

With regard to RPE during work (on a scale from 6 to 20), no change was observed for either of the training groups compared to the control group neither after 12 weeks (the soccer group: 0.3, 95% CI, −1.2, 1.9, P = 0.69/the Zumba group: 0.4, 95% CI, −1.1, 1.9, P = 0.60) or 40 weeks (the soccer group: −0.2, 95% CI, −1.9, 1.6, P = 0.86/the Zumba group: 0.0, 95% CI, −1.6, 1.7, P = 0.99; see S2 Table).

The ANCOVA-analyses without BMI as a covariate revealed similar intervention effects as when the model was adjusted for BMI which indicate that BMI had no confounding effect on the outcome variables in the present study. The per-protocol analyses revealed similar results as the ITT-analyses. Moreover, the Bonferroni method correcting for multiple statistical tests confirmed the statistical significant findings in the study.

## Discussion

To our knowledge, the present study is the first workplace randomized controlled study investigating short- and long-term effects of soccer and Zumba on muscle pain intensity and duration, work ability and RPE during work. The main findings were improvements in pain intensity and duration in the neck-shoulder region in both training groups compared to the control group. Furthermore, the Zumba group showed a tendency for improvement in work ability after 12 weeks compared to the control group. No intervention effects were observed in RPE during work.

In the present study, the soccer group significantly reduced pain intensity in the neck-shoulder region both after 12 weeks (−69%) and 40 weeks (−62%) weeks compared to the control group. These relatively considerable improvements were observed when including all participants in the soccer group (i.e. both those with and without pain at baseline) in an intention-to-treat analyses. This finding supports that soccer leads to reductions in pain intensity of the neck-shoulder region among female hospital employees. In the Zumba group, a similarly significant reduction in neck-shoulder pain intensity was observed after 12 weeks (−62%), with no significant difference after 40 weeks (−26%, P = 0.13). These findings were supported by calculations of effect-sizes (beta squared values>0.09), which may indicate moderate clinical effects from both intervention groups after 12 weeks. Furthermore, the findings are in accordance with previous workplace health promotion studies observing positive effects on neck-shoulder pain intensity from strengthening and/or stretching exercises among different occupational groups [22]–[25].

After 40 weeks, both intervention groups significantly decreased the number of days with neck-shoulder pain compared to the control group, i.e. −16.4 (P<0.02) and −16.6 (P<0.01) days in the soccer and the Zumba group respectively. The potential causes of the positive effects on neck-shoulder pain intensity and duration from soccer and Zumba training may be an increase of anti-inflammatory serum biomarkers [26], [27], beneficial stimulation of sensory and effective pain networks in the central nervous system [28], improved vascular adaption and blood flow to neck-shoulder muscles [29], [30], or general improvements in physical capacity leading to reductions of the relative physical workload [13]. No intervention effects were observed in lower back pain, which is in accordance to previous workplace physical intervention studies [4], [31]–[33]. However, the explanation for the lack of effect on lower back pain is unknown.

After 12 weeks, the Zumba group showed a tendency for improvement in work ability compared to the control group (7%, P = 0.14), with no such change in the soccer group (4%, P = 0.84). Apart from a recent intervention study demonstrating that strength training may prevent deterioration of work ability [34], no previous randomized controlled workplace physical training intervention study has observed improvements in work ability after 12 weeks. The present study did not reveal any intervention effects on work ability after 40 weeks. The improvement in work ability in the Zumba group after 12 weeks may be due the corresponding ∼7% improvement in aerobic capacity compared to the control group [14]. However, as the latter study revealed a similar improvement in aerobic capacity in the soccer group, this may not be the only explanation to the improvement in work ability.

Previous studies have demonstrated a linear relationship between RPE and cardiovascular fitness [35], [36]. Furthermore, it is suggested that RPE provides information on the balance between work demands and the capacity to perform work, and that RPE therefore may be modulated both by lowering physical work demands and by increasing physical capacity [37]. Neither of the intervention groups revealed significant changes in the RPE during work compared to the control group after 12 or 40 weeks. The explanation may be the relatively moderate RPE at work before the intervention (average score of 10 on a scale from 6 to 20). The lack of effect in RPE in the present study is in accordance to a previous workplace physical training intervention study, which also demonstrated improvement in aerobic capacity among construction workers [38]. Hence, apart from one previous workplace physical training intervention study showing improvement in RPE during work among female nurses after 12 month follow-up [32], it may seem that workplace training intervention studies do not improve RPE at work, even in workgroups characterized by high physical work demands.

A strength of the present study was the cluster-randomized controlled design and intention-to-treat evaluation, which is considered to be the gold standard in evaluating interventions [39]. ITT-analyses were considered to be the most adequate statistical analyses method because missing values were not at random. Additional strengths were the high adherence to the training intervention during the first 12 weeks, as well as the relatively high response rates to the questionnaire at baseline (95%), after 12 (84%) and 40 (64%) weeks. These response rates were calculated on the basis of the number of participants who were randomized after the baseline test (N = 107).

Limitations of the study are the relatively moderate adherence to training in both intervention groups between week 12 and 40. Despite of several significant results, the relatively high drop-out rate towards the 40 weeks may have influenced on the statistical power. Furthermore, because we only have information about 5 of the 553 female employees who did not participate in the study, we do not know if the participants are representative for all invited workers at the hospital. In this context, there are some uncertainties with regard to the generalizability. However, in general we consider the employees in the present Hospital to be representative to other Hospitals in Norway or abroad. Another possible bias is the lack of control for leisure time physical activities taking place outside the intervention. Seasonal variations in physical activity patterns due to climatic characteristics in areas north of the Arctic Circle may be a confounder to the study because it could lead to differences in physical activity pattern between the control group and the intervention groups. Moreover, self-reported data represents a potential bias in the present study. However, we have no reason to believe that the intervention groups respond different to the questions compared to the control group.

## Conclusion

The present study indicates that workplace initiated twice-weekly soccer and Zumba training reduce pain intensity in the neck-shoulder region after only 12 weeks, whereas only the soccer group revealed improvement in pain intensity in the neck-shoulder region after 40 weeks. Moreover, both intervention groups reduced the duration of pain in neck-shoulder region after 40 weeks. Furthermore, the Zumba group tended to improve work ability after 12 weeks.

## Supporting Information

S1 Figure Intervention flow. Flow chart on employee recruitment and reach.
(TIF)

S1 Table Descriptive information of the study population at baseline. The table presents age, anthropometry, muscle pain intensity (on a scale from 0 to 10) and duration of muscle pain (number of days during the past 3 months) in the neck-shoulder and lower back region, work ability (on a scale from 0 to 10), and rating of perceived physical exertion (RPE) at work (on a scale from 6 to 20) in the soccer group (n = 35), the Zumba group (n = 34) and the control group (n = 34).
(DOCX)

S2 Table Intervention effects. Changes in muscle pain intensity in the neck-shoulder and lower back region (on a scale from 0 to 10), duration of muscle pain (number of days with pain during the past 3 months), work ability (on a scale from 0 to 10), and rating of perceived physical exertion (RPE) at work (on a scale from 6 to 20) based on ANCOVA analyses with intention-to-treat in the soccer group (n = 35), the Zumba group (n = 34) and the control group (n = 34). The changes and respective P-values illustrate differences between each of the intervention group and the control group from baseline to 12 and 40 weeks follow-up, respectively.
(DOCX)

S1 Checklist Consort 2010 checklist.
(DOC)

S1 Protocol Project approval from the Regional Committees for Medical and Health Research Ethics (Norway).
(PDF)

## References

[1] Warming S, Ebbehoj NE, Wiese N, Larsen LH, Duckert J, et al (2008) Little effect of transfer technique instruction and physical fitness training in reducing low back pain among nurses: a cluster randomised intervention study. Ergonomics 51:1530–1548. 18803093 10.1080/00140130802238606

[2] Steege LM, Drake DA, Olivas M, Mazza G (2013) Evaluation of physically and mentally fatiguing tasks and sources of fatigue as reported by registered nurses. J Nurs Manag [Epub ahead of print]. 10.1111/jonm.12112 23848464

[3] Rongen A, Robroek SJ, van der Heijden BI, Schouteten R, Hasselhorn HM, et al. (2013) Influence of work-related characteristics and work ability on changing employer or leaving the profession among nursing staff. J Nurs Manag [Epub ahead of print]. 10.1111/jonm.12066 23941401

[4] Christensen JR, Faber A, Ekner D, Overgaard K, Holtermann A, et al (2011) Diet, physical exercise and cognitive behavioral training as a combined workplace based intervention to reduce body weight and increase physical capacity in health care workers - a randomized controlled trial. BMC Public Health 11:671. 21871113 10.1186/1471-2458-11-671 PMC3175468

[5] Arvidson E, Borjesson M, Ahlborg G Jr, Lindegard A, Jonsdottir IH (2013) The level of leisure time physical activity is associated with work ability-a cross sectional and prospective study of health care workers. BMC Public Health 13:855. 24044699 10.1186/1471-2458-13-855 PMC3848003

[6] Fischer FM, Borges F, Rotenberg L, Latorre MR, Soares NS, et al (2005) Work ability of health care shift workers: what matters? Chronobiol Int 23:1165–1179. 10.1080/07420520601065083 17190703

[7] Fronteira I, Ferrinho P (2011) Do nurses have a different physical health profile? A systematic review of experimental and observational studies on nurses’ physical health. J Clin Nurs 20:2404–2424. 21752130 10.1111/j.1365-2702.2011.03721.x

[8] Lee S-J, Faucett J, Gillen M, Krause N (2013) Musculoskeletal pain among critical-care nurses by availability and use of patient lifting equipment: An analysis of cross-sectional survey data. Int J Nurs Stud 50:1648–1657. 23648391 10.1016/j.ijnurstu.2013.03.010

[9] Sherehiy B, Karwowski W, Marek T (2004) Relationship between risk factors and musculoskeletal disorders in the nursing profession: A systematic review. Occupational Ergonomics 4:241–279.

[10] Eriksen W, Bruusgaard D, Knardahl S (2003) Work factors as predictors of sickness absence: a three month prospective study of nurses’ aides. Occup Environ Med 60:271–278. 12660375 10.1136/oem.60.4.271 PMC1740518

[11] Eriksen W, Bruusgaard D (2002) Physical leisure-time activities and long-term sick leave: a 15-month prospective study of nurses’ aides. J Occup Environ Med 44:530–538. 12085479 10.1097/00043764-200206000-00014

[12] Strijk JE, Proper KI, van Stralen MM, Wijngaard P, van Mechelen W, et al (2011) The role of work ability in the relationship between aerobic capacity and sick leave: a mediation analysis. Occup Environ Med 68:753–758. 21330573 10.1136/oem.2010.057646

[13] Holtermann A, Jorgensen MB, Gram B, Christensen JR, Faber A, et al (2010) Worksite interventions for preventing physical deterioration among employees in job-groups with high physical work demands: background, design and conceptual model of FINALE. BMC Public Health 10:120. 20214807 10.1186/1471-2458-10-120 PMC2841104

[14] Barene S, Krustrup P, Jackman SR, Brekke OL, Holtermann A (2013) Do soccer and Zumba exercise improve fitness and indicators of health among female hospital employees? A 12-week RCT. Scand J Med Sci Sports: [Epub ahead of print]. 10.1111/sms.12138 24151956

[15] Christensen JR, Overgaard K, Hansen K, Sogaard K, Holtermann A (2013) Effects on presenteeism and absenteeism from a 1-year workplace randomized controlled trial among health care workers. J Occup Environ Med 55:1186–1190. 24064773 10.1097/JOM.0b013e31829b2816

[16] Krustrup P, Hansen PR, Randers MB, Nybo L, Martone D, et al (2010) Beneficial effects of recreational football on the cardiovascular risk profile in untrained premenopausal women. Scand J Med Sci Sports 20 Suppl 1: 40–49. 10.1111/j.1600-0838.2010.01110.x 20210906

[17] Luettgen M, Foster C, Doberstein S, Mikat R, Porcari J (2012) ZUMBA (R): Is the “fitness-party” a good workout? J Sport Sci Med 11:357–358. PMC3737860 24137072

[18] Barene S, Krustrup P, Brekke OL, Holtermann A (2014) Soccer and Zumba as health-promoting activities among female hospital employees: a 40-weeks cluster randomised intervention study. J Sports Sci 32:1539–1549. 24720526 10.1080/02640414.2014.906043

[19] Kuorinka I, Jonsson B, Kilbom A, Vinterberg H, Biering-Sorensen F, et al (1987) Standardised Nordic questionnaires for the analysis of musculoskeletal symptoms. Appl Ergon 18:233–237. 15676628 10.1016/0003-6870(87)90010-x

[20] Borg G (1970) Perceived exertion as an indicator of somatic stress. Scand J Rehabil Med 2:92–98. 5523831

[21] Antes G (2010) The new CONSORT statement. BMJ 340:c1432. 20332507 10.1136/bmj.c1432

[22] Pedersen MT, Andersen CH, Zebis MK, Sjogaard G, Andersen LL (2013) Implementation of specific strength training among industrial laboratory technicians: long-term effects on back, neck and upper extremity pain. BMC Musculoskelet Disord 14:287. 24106771 10.1186/1471-2474-14-287 PMC3851840

[23] Ylinen J, Takala EP, Nykanen M, Hakkinen A, Malkia E, et al (2003) Active neck muscle training in the treatment of chronic neck pain in women: a randomized controlled trial. JAMA 289:2509–2516. 12759322 10.1001/jama.289.19.2509

[24] Ylinen JJ, Hakkinen AH, Takala EP, Nykanen MJ, Kautiainen HJ, et al (2006) Effects of neck muscle training in women with chronic neck pain: one-year follow-up study. J Strength Cond Res 20:6–13. 16503693 10.1519/R-17274.1

[25] Andersen LL, Christensen KB, Holtermann A, Poulsen OM, Sjogaard G, et al (2010) Effect of physical exercise interventions on musculoskeletal pain in all body regions among office workers: a one-year randomized controlled trial. Man Ther 15:100–104. 19716742 10.1016/j.math.2009.08.004

[26] Ortega E, Garcia JJ, Bote ME, Martin-Cordero L, Escalante Y, et al (2009) Exercise in fibromyalgia and related inflammatory disorders: known effects and unknown chances. Exerc Immunol Rev 15:42–65. 19957871

[27] Strohacker K, Wing RR, McCaffery JM (2013) Contributions of body mass index and exercise habits on inflammatory markers: a cohort study of middle-aged adults living in the USA. BMJ open 3. 10.1136/bmjopen-2013-002623 PMC3657650 23793684

[28] Ellingson LD, Colbert LH, Cook DB (2012) Physical activity is related to pain sensitivity in healthy women. Med Sci Sports Exerc 44:1401–1406. 22217571 10.1249/MSS.0b013e318248f648

[29] Strom V, Knardahl S, Stanghelle JK, Roe C (2009) Pain induced by a single simulated office-work session: time course and association with muscle blood flux and muscle activity. Eur J Pain 13:843–852. 19083247 10.1016/j.ejpain.2008.11.003

[30] Sogaard K, Blangsted AK, Nielsen PK, Hansen L, Andersen LL, et al (2012) Changed activation, oxygenation, and pain response of chronically painful muscles to repetitive work after training interventions: a randomized controlled trial. Eur J Appl Physiol 112:173–181. 21512799 10.1007/s00421-011-1964-6 PMC3253274

[31] Yu W, Yu IT, Wang X, Li Z, Wan S, et al (2013) Effectiveness of participatory training for prevention of musculoskeletal disorders: a randomized controlled trial. Int Arch Occup Environ Health 86:431–440. 22544420 10.1007/s00420-012-0775-3

[32] Horneij E, Hemborg B, Jensen I, Ekdahl C (2001) No significant differences between intervention programmes on neck, shoulder and low back pain: a prospective randomized study among home-care personnel. J Rehabil Med 33:170–176. 11506215

[33] Coury HJ, Moreira RF, Dias NB (2009) Evaluation of the effectiveness of workplace exercise in controlling neck, shoulder and low back pain: a systematic review. Rev Bras Fisioter 13:461–479.

[34] Sundstrup E, Jakobsen MD, Brandt M, Jay K, Persson R, et al (2014) Workplace strength training prevents deterioration of work ability among workers with chronic pain and work disability: a randomized controlled trial. Scand J Work Environ Health 40:244–251. 24535014 10.5271/sjweh.3419

[35] Borg GA (1982) Psychophysical bases of perceived exertion. Med Sci Sports Exerc 14:377–381. 7154893

[36] Borg G, Linderholm H (1967) Perceived exertion and pulse rate during graded exercise in various age groups. Acta Med Scand 181:194–206.

[37] Andersen LL, Clausen T, Persson R, Holtermann A (2012) Perceived physical exertion during healthcare work and prognosis for recovery from long-term pain in different body regions: Prospective cohort study. BMC Musculoskelet Disord 13:253. 23253634 10.1186/1471-2474-13-253 PMC3540008

[38] Gram B, Holtermann A, Sogaard K, Sjogaard G (2012) Effect of individualized worksite exercise training on aerobic capacity and muscle strength among construction workers–a randomized controlled intervention study. Scand J Work Environ Health 38:467–475. 22057836 10.5271/sjweh.3260

[39] Schulz KF, Altman DG, Moher D (2010) CONSORT 2010 Statement: Updated guidelines for reporting parallel group randomised trials. J Clin Epidemiol 63:834–840. 20346629 10.1016/j.jclinepi.2010.02.005

