# Supplementary figures and images for: Effects of the Workplace Health Promotion Activities Soccer and Zumba on Muscle Pain, Work Ability and Perceived Physical Exertion among Female Hospital Employees
Source: PLoS One. 2014 Dec 10;9(12):e115059. doi: 10.1371/journal.pone.0115059 (PMC4262471; doi:10.1371/journal.pone.0115059)

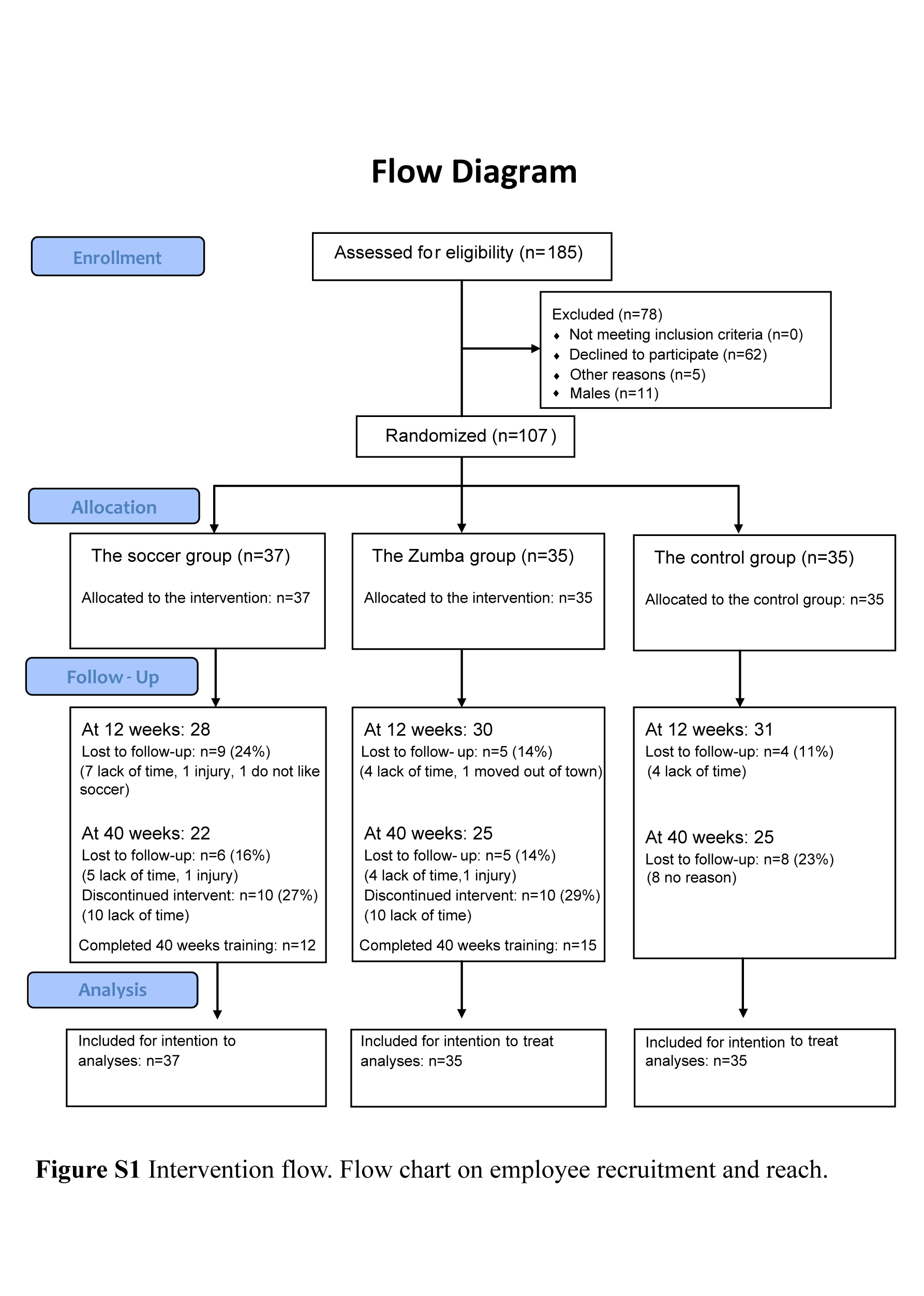

Supplement: S1 Figure — Intervention flow. Flow chart on employee recruitment and reach. (TIF) [file pone.0115059.s001.tif]
