# Supplementary material for: Effects of the Workplace Health Promotion Activities Soccer and Zumba on Muscle Pain, Work Ability and Perceived Physical Exertion among Female Hospital Employees
Source: PLoS One. 2014 Dec 10;9(12):e115059. doi: 10.1371/journal.pone.0115059 (PMC4262471; doi:10.1371/journal.pone.0115059)
Supplement: S1 Table — Descriptive information of the study population at baseline. The table presents age, anthropometry, muscle pain intensity (on a scale from 0 to 10) and duration of muscle pain (number of days during the past 3 months) in the neck-shoulder and lower back region, work ability (on a scale from 0 to 10), and rating of perceived physical exertion (RPE) at work (on a scale from 6 to 20) in the soccer group (n = 35), the Zumba group (n = 34) and the control group (n = 34). (DOCX) [file pone.0115059.s002.docx]

**Table S1.**

**Descriptive information of the study population at baseline.** The table presents age, anthropometry, muscle pain intensity (on a scale from 0 to 10) and duration of muscle pain (number of days during the past 3 months) in the neck-shoulder and lower back region, work ability (on a scale from 0 to 10), and rating of perceived physical exertion (RPE) at work (on a scale from 6 to 20) in the soccer group (n=35), the Zumba group (n=34) and the control group (n=34).

| **Characteristics** | **Soccer group** | | **Zumba group** | | **Control group** | | **Total** | |  |
| --- | --- | --- | --- | --- | --- | --- | --- | --- | --- |
|  | Mean | SD | Mean | SD | Mean | SD | Mean | SD |  |
| Age (years) | 44.4 | 8.6 | 45.9 | 9.6 | 47.4 | 9.5 | 45.9 | 9.2 |  |
| Weight (kg) | 70.0 | 8.7 | 71.0 | 8.2 | 71.8 | 11.4 | 70.9 | 9.5 |  |
| Height (cm) | 167.6 | 5.3 | 167.5 | 6.2 | 166.1 | 5.8 | 167.1 | 5.8 |  |
| Body mass index (kg/m^2^) | 24.9 | 3.1 | 25.3 | 2.7 | 26.0 | 3.6 | 25.4 | 3.1 |  |
| Work seniority (months) | 90 | 65 | 77 | 62 | 69 | 65 | 78 | 64 |  |
| Working hours per week | 35.6 | 5.1 | 34.2 | 6.4 | 37.4 | 4.1 | 35.8 | 5.4 |  |
| Pain intensity (0-10) |  |  |  |  |  |  |  |  |  |
| Neck-shoulder region | 2.6 | 2.2 | 1.7 | 2.1 | 1.8 | 2.0 | 2.0 | 2.1 |  |
| Lower back region | 1.6 | 1.9 | 1.5 | 2.1 | 1.4 | 1.8 | 1.5 | 1.9 |  |
| Pain duration (number of days during the past 3 months) |  |  |  |  |  |  |  |  |  |
| Neck-shoulder region | 22.0 | 29.7 | 14.2 | 24.6 | 17.6 | 23.1 | 18.0 | 26.0 |  |
| Lower back region | 7.1 | 17.6 | 8.3 | 17.1 | 11.5 | 22.7 | 8.9 | 19.2 |  |
| Work ability (0-10) | 7.3 | 1.3 | 7.2 | 1.4 | 7.8 | 1.1 | 7.4 | 1.3 |  |
| RPE during work (6-20) | 10.0 | 3.4 | 11.3 | 3.5 | 9.0 | 2.9 | 10.0 | 3.4 |  |
|  |  |  |  |  |  |  |  |  | |
