# Supplementary material for: Effects of the Workplace Health Promotion Activities Soccer and Zumba on Muscle Pain, Work Ability and Perceived Physical Exertion among Female Hospital Employees
Source: PLoS One. 2014 Dec 10;9(12):e115059. doi: 10.1371/journal.pone.0115059 (PMC4262471; doi:10.1371/journal.pone.0115059)
Supplement: S2 Table — Intervention effects. Changes in muscle pain intensity in the neck-shoulder and lower back region (on a scale from 0 to 10), duration of muscle pain (number of days with pain during the past 3 months), work ability (on a scale from 0 to 10), and rating of perceived physical exertion (RPE) at work (on a scale from 6 to 20) based on ANCOVA analyses with intention-to-treat in the soccer group (n = 35), the Zumba group (n = 34) and the control group (n = 34). The changes and respective P-values illustrate differences between each of the intervention group and the control group from baseline to 12 and 40 weeks follow-up, respectively. (DOCX) [file pone.0115059.s003.docx]

**Table S2.**

**Intervention effects.** Changes in muscle pain intensity in the neck-shoulder and lower back region (on a scale from 0 to 10), duration of muscle pain (number of days with pain during the past 3 months), work ability (on a scale from 0 to 10), and rating of perceived physical exertion (RPE) at work (on a scale from 6 to 20) based on ANCOVA analyses with intention-to-treat in the soccer group (n=35), the Zumba group (n=34) and the control group (n=34). The changes and respective P-values illustrate differences between each of the intervention group and the control group from baseline to 12 and 40 weeks follow-up, respectively.

| **Characteristics** | **Soccer group** | | | **Zumba group** | | |
| --- | --- | --- | --- | --- | --- | --- |
|  | Diff. | 95% Cl | Sig. | Diff. | 95% Cl | Sig. |
| Pain intensity (0-10) |  |  |  |  |  |  |
| Neck-shoulder region |  |  |  |  |  |  |
| 0-12 weeks | -1.9 | -3.0 to -0.8 | 0.00 | -1.3 | -2.3 to -0.3 | 0.01 |
| 0-40 weeks | -1.9 | -3.2 to -0.7 | 0.00 | -0.9 | -2.0 to 0.3 | 0.13 |
| Lower back region (0-10) |  |  |  |  |  |  |
| 0-12 weeks | -0.1 | -1.1 to 0.8 | 0.82 | 0.2 | -0.7 to 1.1 | 0.69 |
| 0-40 weeks | -0.5 | -1.5 to 0.4 | 0.27 | -0.4 | -1.3 to 0.5 | 0.41 |
| Duration of pain (number of days during the past 3 months) |  |  |  |  |  |  |
| Neck-shoulder region |  |  |  |  |  |  |
| 0-12 weeks | -9.2 | -20.0 to 1.6 | 0.10 | -7.0 | -17.2 to 3.1 | 0.17 |
| 0-40 weeks | -16.4 | -29.6 to -3.2 | 0.02 | -16.6 | -28.9 to -4.2 | 0.01 |
| Lower back region |  |  |  |  |  |  |
| 0-12 weeks | 5.8 | -4.3 to 15.9 | 0.26 | 3.0 | -6.3 to 12.3 | 0.52 |
| 0-40 weeks | -7.0 | -18.8 to 4.8 | 0.24 | -6.8 | -17.5 to 3.8 | 0.21 |
| Work ability (0-10) |  |  |  |  |  |  |
| 0-12 weeks | 0.1 | -0.6 to 0.8 | 0.84 | 0.5 | -0.2 to 1.1 | 0.14 |
| 0-40 weeks | -0.0 | -0.7 to 0.7 | 0.91 | 0.3 | -0.4 to 0.9 | 0.44 |
| RPE during work (6-20) |  |  |  |  |  |  |
| 0-12 weeks | 0.3 | -1.2 to 1.9 | 0.69 | 0.4 | -1.1 to 1.9 | 0.60 |
| 0-40 weeks | -0.2 | -1.9 to 1.6 | 0.86 | 0.0 | -1.6 to 1.7 | 0.99 |
