# Supplementary material for: Effects of the Workplace Health Promotion Activities Soccer and Zumba on Muscle Pain, Work Ability and Perceived Physical Exertion among Female Hospital Employees
Source: PLoS One. 2014 Dec 10;9(12):e115059. doi: 10.1371/journal.pone.0115059 (PMC4262471; doi:10.1371/journal.pone.0115059)
Supplement: S1 Protocol — Project approval from the Regional Committees for Medical and Health Research Ethics (Norway). (PDF) [file pone.0115059.s005.pdf]

Form: Project Approval

Forms received 02/09/2010 in vigorous - scissors portal for the Regional Committees for Medical and Health Research Ethics (REK) on; helseforskning.etikkom.no

2010/2385-1

Summary

1. **General information**

   A. Project Title
   Project Title: Soccer, Zumba and health
   Scientific title: Physiological
   Project : Svein Barene
   Research coordinator: University of Nordland, Norway (UIN)
   Initiator : Project or Research Manager (Research Contributions)
   Education Project:
   Study : Study of professional knowledge
   Level : PhD
   Norwegian title Soccer, Zumba and health
   Scientific title: Physiological, psychological and sociological health effects from soccer and Zumba, respectively, in a worksite .

   B. Project leader
   Name: Svein Barene
   Degree: Master of Science
   Clinical expertise:
   Position: PhD student
   Main work: UIN
   Work Address: Centre for Performance
   Zip 8049
   Location: Bodø, Norway
   Phone: 75517564
   phone:
   E - mail address: svein.barene@hibo.no

   C. Research responsible
   Research coordinator is: Institution or other legal person
   Institutional / legal person: UIN
   Street Address / PO Box: v/Centre for Performance
   Zip: 8049
   Location: Bodø
   Contact: Svein Barene
   Position: PhD student
   Phone: 75517564

Mobile Phone: 95258477
E - mail address: svein.barene@hibo.no

D. Project location
Initiator of the project: Project or Research Manager (Research Contributions)
Cooperation with foreign countries: Yes
Part of an international multicenter trial: Yes
Independent data collection abroad:
Education Project/PhD project: Yes
Study: Study of professional knowledge
Level: PhD
Related research: Yes
Project Title: Soccer and Health
Alternatively REK No:
Any remaining case number in REK:

E. Researchers
Name: Jan Selmer Methi
Position: Associate professor
Institution: UIN
Academic degree: PhD
Project Role: Supervisor

Name: Peter Krustrup
Position: Lecturer
Institution: University of Copenhagen, Department of Sports (Denmark)
Academic degree: PhD
Project Role: Supervisor

Name: Andreas Holtermann
Position: Senior Researcher
Affiliation: National Research Centre for the Working Environment, Copenhagen (Denmark)
Academic degree: PhD
Project Role: Supervisor

Name: Ole-Lars Brekke
Position: Consultant /1. Assistant Professor
Institution: Nordland Hospital, Department of Laboratory Medicine/University of Tromsø (Norway)
Faculty of Health Sciences, Institute of Medical Biology
Degree: Dr.med
Project Role: Collaborator

2. Project Information

A. Background and Purpose

Project description by the Project Manager:
At the University of Copenhagen previous studies have shown that soccer is more health beneficial compared with for example running and strength training. In this study, we want to conduct an intervention study among a working population in Bodø, i.e. 12-week training period of soccer and the Latin- inspired dance fitness Zumba, respectively. The different training groups will be tested for physiological parameters before and after the training period in order to compare the effects of the two training forms. In addition to the two intervention groups we will establish a control group who will only participate in the tests. Furthermore, we wish to highlight experiences that occur among employees through participation in training activities outside working hours.

B. Research Data

New health information: Yes
Specify the types of health information: VO2 measurements, body fat percentage, bone density, blood samples (lipids-/lactate measurements), blood pressure measurements
Human biological material: Yes
Data collection: The material will be collected during the project period.

C. Research Methods

The project is: Both quantitative and qualitative
Intervention: Yes
Clinical examination: Yes
Questionnaire: Yes
Interview: Yes
Audio recording: Yes
Observation: Yes
Film / Video: Yes
Photograph: Yes

Academic and scientific justification for the choice of method:
In an activity-theoretical perspective, one can see any phenomenon from a quantitative and a qualitative conditions (Marx , 2008). In this study, we want to use both quantitative and qualitative methods. The quantitative data has its own form of knowledge, and often expressed in proportional sizes. They reproduce a greatly reduced relationship that exist in reality, but are not irrelevant. It is possible to understand that a man is subjected to a special controllable physical activity may have changed his muscle mass and oxygen consumption for given conditions. However, it is not possible to deduce from this that the wellness increases. This requires a qualitative approach. In the qualitative approach, the quantitative data lie in the background. The qualitative study will be looked at how physical activity turns out, and is expressed by the participants themselves. It is their experience – and the researchers’ understanding of it, that gets attention. For a coach, we assume that both of these conditions are important to understand. The quantitative data provides some facts about the effects that come out of these activities, but provides no deeper understanding of what they actually represent. In this study, we will try to look at the activities from a phenomenological perspective, and attempt to create

understanding (from my position as coach) by reflections upon my observations. We will divide this work into three phases:

Phase 1: The initial phase (before intervention).
Method: Interview.
In this phase we will randomly select 5-6 subjects from each intervention group to interview.

Phase 2: Intermediate phase (during the intervention).
Method: Questionnaire and field interviews.
Subjects will initial to the training be given a questionnaire consisting of four new closed questions which are prepared on the basis of field interviews in the previous exercise. During the training sessions, I will conduct field interviews with 2-4 subjects who are based in spontaneous situations that arise.

Phase 3: The final phase (after intervention).
Method: Interview. The same subjects who were interviewed in Phase 1 will at this stage be invited to a new interview, which will focus on the experience and lessons learned through participation in the respective intervention groups.

The quantitative data will be conducted in a controlled, cluster - randomized design, in which groups of employees who have a work community, e.g. the same working hours, will be drawn randomly to one of three exercise groups;

Group 1: Soccer (3 x 60 min) - 30 people playing soccer 2 x 30 minutes three sessions per week for 12 weeks. The soccer training will be organized by dividing into teams consisting of 4-8 players, and will be played on small courts either outside or inside. The training intensity is measured with heart rate monitors and movements registered by GPS or time-motion .

Group 2: Zumba (3 x 60 minutes) - 30 employees will be perform Zumba at “Friskhuset” 60 minutes three sessions per week for 12 weeks. The Zumba training will be conducted with certified instructor in a dance-room. The training intensity is measured with heart rate monitors.

Group 3: Control group: - 30 employees receive no training, but will be offered health consultation based on information from the physiological tests.

Health indicators :
Health effects: Body weight, body fat percentage, and bone density, blood pressure, blood glucose, cholesterol, physical capacity (including aerobic endurance, strength, flexibility, balance ability and reaction time).
Effects on absenteeism, effects on employees experienced psychological work environment.

D. Selection
General Population: Yes
Control group (s): Yes

Specify who should be included in the control group(s): A random sample from the companies involved.
Justify the choice of control group(s): In order to have a basis for comparison in relation to any effects of training in the two training groups.

E. Scope
Norway: 80-130
Explain and justify the project scope: Refer to "Technical and scientific justification for the choice of data and method " and the attached protocol.

2E. Biobank

2C. Biobank
A.
New specific research biobank: Yes
Biobank name: Soccer, Zumba and health

3. Consent and Privacy

A.
B.
Consent obtained: Yes
For what data should consent be obtained? All
Specifically actively informed written consent: Yes
Elaborate how to ensure a free and informed consent and explain any deviations from recommended procedures: It will be held an information meeting with the employees of the company, and it is essential that all participation in the intervention study is voluntary.

C.

4. Ethical assessment of the advantages and disadvantages

A. Benefits
All participants: Yes
Specify the advantages: Participation in one of the training groups will lead to increased physical activity for participants, and you will most likely achieve better physical capacity.
Groups of people: Yes
Specify which groups: training groups, staff groups engaged in joint training activities that represent something other than the work you normally do together.
Specify the advantages: Interaction and cooperation which are perceived as enjoyable. Increases of the community wellness, team -building.
Society: Yes
Specify the advantages: Focuses on physical activity and health in a community perspective.
Science: Yes

Specify the advantages: Documentation of physiological, psychological and sociological effects of two different training methods.

B. Disadvantages
Each project participant: Yes
Enter the disadvantages: Can cause minor sport related injuries, such as ankle twisting, etc., but from studies in Denmark, this occurs to a small extent. There will obviously be a discomfort associated with venipuncture, but this is considered a minimal problem and without risk of complications or accidents. Running test will of course entail degree of fatigue towards the end.

C. Measures
Elaborate the safety of the participants in the research project: Participants will be closely monitored throughout the intervention period, and the security of training will be addressed in a proper manner. In the event of any damage, the involved will be offered emergency medical treatment. Warm-up will be conducted so that participants are well prepared to prevent injuries, etc.

D. Ethical considerations
Describe your trade-off between advantages and disadvantages and give your reasons for why you believes it is prudent to undertake the project:
It is not considered to be any risks of participation in the project beyond regular physical activity time .

5. Safety, interests and publication

A. Personally identifiable information
Personal information recorded in the project is directly personally identifiable: Yes
Name, address, or date of birth: Yes
11- digit identity number: Yes

B. Internal Control and Security
Physically isolated computer related activities: Yes
Video recording / photography: Yes
On personal computer (PC): Yes
Audio recording: Yes
On PC: Yes
Manual / Paper: Yes
Linking Key and data stored separately from each other: Yes
Background information (place of residence, occupation , etc.) are kept separate from other information: Yes
Password Protected storage: Yes
Incarcerated storage: Yes
Explain in more detail how personally identifiable information is protected from unauthorized access: All data will be locked in a safe for safe storage.

C. Insurance Coverage for participants

Insurance is considered unnecessary: Yes
Explain why it is considered unnecessary insurance for research participants:

D. Assessment of other agencies

Private institution: Yes

E. Interests

Funding: The project is a cooperation between Bodø, Nordland Hospital, Friskhuset, University of Copenhagen, and the National Centre for our work environment (Copenhagen).
Remuneration to the institution:
Fees manager/staff:
Compensation for research participants:
Any conflicts of interest for the project manager/staff: No, there are no such constraints.

F. Publication

Explain how the results should be made publicly available:
The results will be published in recognized international journals.

G. Public disclosure

H. Timeframe

Project Start date: 02/09/2010
Project End Date: 19.11.2015
After the end of year data de-identified: Yes
Explain further handling of data after project completion: Data will be stored in locked safes.

6. Appendix

1 Protokoll_SBA020910.pdf - Research Protocol - 03.09.10
2 Spørreskjema_020910.pdf - Questionnaire - 03.09.10
3 Samtykkeskjema_SBA020910.pdf - Consent form - 03.09.10
4 Intervjuguide_010910.pdf - Interview Guide - 02.09.10
5 Infoskriv_fysisk activity and arbeid.pdf - Request for participation - 02.09.10
6 screening_230610.pdf - Sreeningskjema - 02.09.10

7. Legal notice

A.

I declare that the project will be conducted in accordance with applicable laws, regulations and Guidelines: Yes
I declare that the project will be conducted in accordance with the information provided in this application: Yes
I declare that the project will be conducted in accordance with any conditions of approval given by the IEC or other authorities: Yes
